# Abiotic stress QTL in lettuce crop–wild hybrids: comparing greenhouse and field experiments

**DOI:** 10.1002/ece3.1060

**Published:** 2014-05-17

**Authors:** Yorike Hartman, Danny A P Hooftman, Brigitte Uwimana, M Eric Schranz, Clemens C M van de Wiel, Marinus J M Smulders, Richard G F Visser, Richard W Michelmore, Peter H van Tienderen

**Affiliations:** 1 Institute for Biodiversity and Ecosystem Dynamics, Universiteit van AmsterdamAmsterdam, The Netherlands; 2 NERC, Centre for Ecology and HydrologyWallingford, UK; 3 Wageningen UR Plant Breeding, Wageningen University and Research CentreWageningen, The Netherlands; 4 Genome Center and Department of Plant Sciences, University of CaliforniaDavis, California

**Keywords:** Abiotic stress, crop–wild hybrids, environmental risk assessment, *Lactuca*, QTL, Transgene

## Abstract

The development of stress-tolerant crops is an increasingly important goal of current crop breeding. A higher abiotic stress tolerance could increase the probability of introgression of genes from crops to wild relatives. This is particularly relevant to the discussion on the risks of new GM crops that may be engineered to increase abiotic stress resistance. We investigated abiotic stress QTL in greenhouse and field experiments in which we subjected recombinant inbred lines from a cross between cultivated *Lactuca sativa* cv. Salinas and its wild relative *L. serriola* to drought, low nutrients, salt stress, and aboveground competition. Aboveground biomass at the end of the rosette stage was used as a proxy for the performance of plants under a particular stress. We detected a mosaic of abiotic stress QTL over the entire genome with little overlap between QTL from different stresses. The two QTL clusters that were identified reflected general growth rather than specific stress responses and colocated with clusters found in earlier studies for leaf shape and flowering time. Genetic correlations across treatments were often higher among different stress treatments within the same experiment (greenhouse or field), than among the same type of stress applied in different experiments. Moreover, the effects of the field stress treatments were more correlated with those of the greenhouse competition treatments than to those of the other greenhouse stress experiments, suggesting that competition rather than abiotic stress is a major factor in the field. In conclusion, the introgression risk of stress tolerance (trans-)genes under field conditions cannot easily be predicted based on genomic background selection patterns from controlled QTL experiments in greenhouses, especially field data will be needed to assess potential (negative) ecological effects of introgression of these transgenes into wild relatives.

## Introduction

Drought, salinization, and other abiotic stresses are major causes of crop loss. These crop losses are expected to increase worldwide due to global warming, leading to a loss of land available for agriculture and a reduction in yields (Cominelli and Tonelli 2010). Increasingly research is focused on developing crops that are resistant or tolerant to abiotic stresses such as drought, salinity, heat, cold, flooding, and nutrient limitation (as reviewed in Bhatnagar-Mathur et al. 2008; Collins et al. 2008; Witcombe et al. 2008). The introduction of stress-tolerant genetically modified (GM) crops could contribute to higher yields under such conditions. At the same time, public and governmental concern about the consequences of transgene escape in the environment has led to stringent policies and elaborate risk assessment strategies (Snow et al. 2005; EFSA 2011). In case a transgene contributes to a higher fitness or competitiveness of the wild relative, it could lead to an increased weediness or the invasion of new habitats (Pilson and Prendeville 2004). Such increased weediness has been observed for several wild relatives that received conventional crop alleles through hybridization (Ellstrand et al. 2013), although negative environmental effects have not yet been identified (Kwit et al. 2011). It has been argued that especially abiotic tolerance transgenes could have potential unwanted effects. For example, acquisition of drought or salt tolerance could expand the typical habitat range of a wild relative growing in such more adverse conditions (Andow and Zwahlen 2006).

Currently, environmental risk assessment (ERA) procedures are performed on a case-by-case basis (EFSA 2011). It is difficult to generate general protocols or guidelines, as data available to evaluate the potential of transgenes to increase invasiveness and/or weediness of wild relatives are still scarce (Kwit et al. 2011; Ellstrand et al. 2013). Given the large research effort to develop new abiotic stress-tolerant transgenic crops, the question arises whether correlative studies such as abiotic stress quantitative trait loci (QTL) can be used to help predict an important step in risk assessment: What is the likelihood of establishment in a population (i.e., introgression) of transgenic constructs after a hybridization event, based on selection pressures on its surrounding genomic region (Stewart et al. 2003)? Only cases for which such introgression is likely would require the subsequent step of risk assessment, namely whether the transgene has an actual fitness impact on the wild population. An important prerequisite of such predictions would be a high generalizability of genomic selection patterns among experiments into the same stress.

The likelihood of introgression of a transgene after a hybridization event does not solely depend on the isolated effect of the gene on the fitness of hybrid individuals. This likelihood also depends on the fitness effects of the genes that are in close linkage with the transgene (Stewart et al. 2003; Chapman and Burke 2006). Introgression is generally on the level of chromosomal segments rather than individual genes; these segments may contain hundreds of genes for a number of generations before they are sufficiently reduced in size through successive recombination events (Stewart et al. 2003). Consequently, crop alleles and transgenes situated in genomic regions under positive selection are more likely to introgress into the wild population than when they are in genomic regions under negative selection (Gressel 1999; Stewart et al. 2003). Thus, the likelihood for introgression may be different for each transgenic event. For example, methodologies as using Agrobacterium or shotgun transformation results in the transgene being inserted at different, not well predefined, locations in the genome. QTL studies allow pinpointing genomic regions under selection that influence traits that may or may not enhance introgression into a wild population (Mauricio 2001). This ability to predict the likelihood of introgression is tightly linked to the reliability of QTL identification, the heritability of the trait, and the power of the experimental design (Beavis 1998).

A number of QTL studies have successfully identified genomic regions under selection for demographic, morphological, and fitness traits in the field, usually identifying a few genomic regions of major effect (Baack et al. 2008; Dechaine et al. 2009; Hartman et al. 2012, 2013a). However, many genes, proteins and metabolic pathways can be involved in stress responses (Vinocur and Altman 2005; Roy et al. 2011). Potentially differences in experimental setup could cause variation in the exact genetic response, which would result in variation in location and effect size of stress-related QTL detected (Collins et al. 2008). The result could be a mosaic of many different genomic regions with small- to medium-sized effects that would make predicting the introgression chances of abiotic stress transgenes more difficult compared with a single region of large effect. Such a mosaic of QTL under general “field” conditions was detected for growth-related traits in lettuce (Hartman et al. 2012, 2013a,b2013b). Here, we will investigate the response to single stress conditions individually.

On the other hand, abiotic stress QTL may coincide with suites of genes that are similarly up- or downregulated in response to multiple stresses, as stress-signaling pathways for different abiotic stresses are connected in regulatory networks with common elements (Knight and Knight 2001). Different stresses may also require the same protective action. For example, plants under cold, drought and salt stress employ similar mechanisms to prevent dehydration (Knight and Knight 2001; Wang et al. 2003). Regulatory genes that can induce such stress responses are the focus of modern transgenic research (Bhatnagar-Mathur et al. 2008; Cominelli and Tonelli 2010). In turn, this may imply that genomic regions of major effect can be found for general abiotic stress responses (or various abiotic stress traits). The existence of such regions of major effects for multiple stresses was suggested in a fully controlled greenhouse experiment with *Lactuca* hybrids (Uwimana et al. 2012b,c2012c). These results are opposite to the mosaic of small- to medium-effect QTL found in field studies, referred to above, in the same species (Hartman et al. 2012, 2013a,b2013b).

A further important factor to consider in evaluating the chances for transgene introgression is the competitive ability of crop–wild hybrid individuals (Chapman and Burke 2006). Campbell and Snow (2007) compiled studies on sunflower, oilseed rape, and radish and showed that for the majority of studies, crop–wild hybrid fitness was reduced under noncompetitive circumstances compared with the wild type. However, under highly competitive conditions, hybrid fitness increased, reducing the difference between hybrid and wild genotypes and thereby increasing the chances of introgression of crop alleles to wild populations (Mercer et al. 2006; Campbell and Snow 2007). Although hybrid fitness as such has been studied in lettuce (Hooftman et al. 2005, 2007), competition and its interaction with abiotic stresses have received little attention.

Our goal of study is to identify genetic regions, which are selected for in the crop–wild model system of lettuce. For practical and environmental safety reasons, we use nontransgenic lines. We will study recombinant inbred lines (RILs) from a cross between the cultivated iceberg lettuce (*Lactuca sativa* cv. Salinas) and its wild relative *L. serriola*. The two parental species have no barriers for hybridization (Koopman et al. 2001). In previous work, we used this RIL population to study neutral morphological traits in the greenhouse, such as leaf morphology, bolting and flowering time, and seed morphology (Hartman et al. 2013b). Moreover, we studied demographic and reproductive traits under natural field environments (Hartman et al. 2012, 2013a) and identified genomic regions under selection. Those studies will be used to interpret our results.

In this study, we set out to test the level of variability in QTL across treatments with a series of experiments in which single or combined stresses were added. Plants were subjected to drought, high salinity, and nutrient limitation in (i) a controlled, noncompetitive greenhouse environment; (ii) a controlled, competitive greenhouse environment; and (iii) a competitive field environment. We focused on aboveground biomass at the end of the rosette stage similar to the moment the crop is normally harvested and therefore pertinent to yield, as an integrative trait to assess the response of the whole plant to stress (Witcombe et al. 2008). Specifically, we addressed the following questions:

1. Which genomic locations in lettuce carry QTL for the response to drought, salinity, nutrient limitation, and intraspecific competition?
2. Can we identify clusters of QTL indicating genomic regions involved in a specific stress or in general abiotic stress tolerance?
3. How does the QTL pattern of abiotic stress without competition compare to the QTL pattern under competition?

We will focus our discussion around whether we identify a few major effect QTL or a mosaic of QTL for multiple stresses. We highlight the implications of our results for ERA procedures of future GM crops.

## Material and Methods

### Plant material

We used an existing recombinant inbred line (RIL) population from a cross between a crop species lettuce (*Lactuca sativa* cv. Salinas) and its wild relative prickly lettuce (*L. serriola* UC96US23). These RILs have been used for various analyses, including Johnson et al. (2000); Argyris et al. (2005), and Zhang et al. (2007). These two closely related and fully interfertile *Lactuca* species (Koopman et al. 2001) show marked differences in phenotype. The *L. serriola* used in the cross to make the RILs (UC96US23) has long serrate leaves that contain white bitter latex. Plants have spines up to 2 mm long on the stem base and leaf midribs. It is considered drought-tolerant with a long taproot with which it can reach water at deep soil layers (Gallardo et al. 1996). In contrast, *L. sativa* cv. Salinas has broad, almost circular, leaves without any spines and a low latex content (de Vries 1997). The crop develops a shallow root system with a short taproot and many lateral branches in the topsoil layer (Jackson 1995). *Lactuca sativa* is therefore adapted to agricultural systems with high inputs of water and nutrients, probably as a consequence of selection during domestication and subsequent breeding (Jackson 1995). In contrast, *L. serriola* mainly occurs in ephemeral ruderal habitats, including roadsides, railways, and construction sites (Lebeda et al. 2001). It is an annual species that flowers in July–August and survives the winter as seed, but sometimes as small rosettes (Yorike Hartman, personal observation). Both species are predominantly selfing, but 1–5% outcrossing rates via insect pollination have been reported (Giannino et al. 2008). Moreover, Uwimana et al. (2012a) inferred from a large population-genetic study using SSR markers that about 7% of the European *L. serriola* plants were offspring of hybridization events between *L. serriola* and *L. sativa*.

### Experimental design

We performed four different abiotic and competition stress experiments in the greenhouse as well as in the field. These included two greenhouse stress experiments, drought/recovery (DR) and salt/nutrient limitation (SN), one greenhouse competition experiment and one field stress experiment in which field conditions were combined with individual stresses.

#### Greenhouse stress experiments

The DR and SN experiments in the absence of competition were performed in February–March 2009 and April–May 2009, respectively (Figure S1). In the DR experiment, plants were grown in pots with soil, whereas in the SN experiment, plants were grown in vermiculite, allowing flushing the substrate with salt solution and more consistency in nutrient limitation. The DR experiment consisted of a control, drought, and recovery treatment and the SN experiment consisted of a control, nutrient limitation, and 100 mmol/L salt treatments (Table 1). From 114 available RILs, we selected a set of 60 lines using MapPop (Vision et al. 2000). MapPop maximizes the number of recombination breakpoints and provided a population with the highest amount of genetic variation. We also included the parent lines. Five replicates per treatment resulted in a total of 930 plants in the DR experiment and 1240 plants in the SN experiment.

**Table 1:** The mean and standard deviation (SD) for the parent lines and the RIL population for all treatments and combined treatments with the drought and field experiments. *T*-test results indicate significance of differences between the parent lines. Broad-sense heritability values (*H*^2^) are given as the percentage of phenotypic variation among RILs. Abbreviations used in Fig. 1 are provided here (Abbr.)

		Crop		Wild		*T*-test			RILs		(*H*^2^)
						
Trait	Abbr.	Mean	SD	Mean	SD	df	T	P	Mean	SD	(%)
Aboveground dry weight (g)											
Salt & nutrient limitation											
Control	DCsn	3.56	0.53	4.17	0.83	8	−1.40	0.199	4.17	0.69	54.7
Salt 100 mmol/L	DSsn	1.85	0.11	1.21	0.20	8	6.15	0.000	1.49	0.30	58.6
Nutrient limitation	DNsn	1.32	0.24	2.27	0.42	8	−4.41	0.002	1.86	0.30	65.5
Increased drought (23 days) and recovery (4 days)											
Control	DCdr	4.59	0.47	4.76	0.84	7	−0.35	0.000	4.74	0.99	39.7
Drought	DDdr	1.61	0.11	1.31	0.05	9	5.28	0.001	1.43	0.14	41.8
Recovery	DRdr	2.01	0.11	1.79	0.19	8	2.18	0.000	1.96	0.21	22.3
Increased competition											
Competition only	DCc	0.35	0.15	0.97	0.47	31	−5.12	0.000	0.88	0.33	51.1
+ Nutrient limitation	DNc	0.48	0.17	0.42	0.09	32	1.24	0.223	0.55	0.15	55.1
+ Salt 100 mmol/L	DSc	0.29	0.14	0.50	0.24	30	−3.07	0.004	0.62	0.19	55.4
+ Drought	DDc	0.39	0.12	0.32	0.14	31	1.55	0.131	0.41	0.14	32.6
Field + stress											
Field only	DCf	5.07	1.59	5.02	1.58	28	0.09	0.931	6.04	2.17	20.0
+ Salt 100 mmol/L	DSf	2.73	1.11	2.61	1.17	20	0.24	0.811	4.10	1.69	19.9
+ Drought	DDf	3.12	1.31	2.60	0.70	22	1.15	0.264	5.13	2.01	17.0
Proportion dry weight (%)											
Salt & nutrient limitation											
Control	PDCsn	4.58	0.64	7.72	1.05	8	−5.72	0.000	6.24	0.62	28.5
Salt 100 mmol/L	PDSsn	7.32	0.56	10.49	0.45	8	−9.84	0.000	8.52	0.35	73.3
Nutrient limitation	PDNsn	6.39	0.43	11.41	0.93	8	−10.9	0.000	9.53	0.70	78.3
Increased drought (23 days) and recovery (4 days)											
Control	PDCdr	7.60	0.46	13.08	0.53	7	−16.2	0.736	10.8	0.99	64.9
Drought	PDDdr	13.1	1.17	16.71	1.37	9	−4.69	0.001	15.5	1.26	64.9
Recovery	PDRdr	7.05	0.50	11.15	0.67	8	−11.0	0.061	9.43	0.66	58.3

##### Experimental variation

We lowered experimental variation within and between RIL families. First, seeds of the RILs and parent lines were germinated in three separate groups based on the results of the germination experiment in Hartman et al. (2013b). On day 1, we started with the slowest germinating group (6 lines), on day 2, with the average group (46 lines and the wild parent) and on day 3, with the fastest germinating group (8 lines and the crop parent). In addition, we assessed all individuals of each RIL at the end of the establishment period and eliminated the five largest and five smallest seedlings, keeping the 15 intermediates for the experiments. To minimize position effects, we randomized the seedlings and later the plants twice a week during the entire seven-week period of the experiment. During the germination period, we randomized trays and Petri dishes, and during the establishment and stress periods, we randomized pots within the blocks. Each treatment had five blocks, and each block contained one individual of all RILs and the parent lines. To monitor stress levels, we also included 25 empty pots per treatment divided over all blocks (Figure S2). In the DR experiment, empty pots were weighed to record the water capacity, whereas in the SN experiment, the electrical conductivity was measured in the plates underneath the pots after flushing the pots to record salt and nutrient stress levels. In addition, temperature and humidity were measured to monitor the stability of greenhouse conditions. For both the DR experiment and the SN experiment, treatment conditions were stable throughout the stress period (Figure S2 and Appendix S1).

##### Germinations and establishment period

Seeds were placed in Petri dishes on filter paper and watered with sterilized water to induce germination. We added a small amount of tetramethyl-thiuram-disulfide powder to prevent the formation of fungi on the seeds. The Petri dishes were placed in a germination cabinet under 16 h of light at 20°C and 8 h dark with 15°C. The germination period lasted 9 days after which seedlings were transplanted to pots of 15 cm diameter with soil (DR) or vermiculite (SN) and grown in the greenhouse under 6 h dark and 18 h light, a minimum of 18°C, under 600 W SON T-Agro lamps generating on average 160 *μ*mol/m²/sec at plant level. This establishment period lasted 9 days.

##### Stress experiments

In the DR experiment, stress was applied by withholding water for 24 days in the drought and recovery treatment, while the control treatment was watered three times a week to keep the soil close to maximum water capacity. After this period, we collected the aboveground biomass for the drought treatment (Figure S1). In the recovery treatment, a subset of plants were watered again. After four more days, we collected both the aboveground biomass of the control and the recovery treatment.

In the SN experiment, the stress period lasted 25 days. Treatments were administered twice a week by flushing the pots containing vermiculite from the top. For the first 4 days, stress levels were built up gradually by flushing the pots twice every day. The control treatment was watered with 1.0 g/l nutrient solution (Scotts, Peters Professional Growth, 20:10:20 NPK). Nutrient limitation was induced by watering without added nutrients. Salt stress was induced with a solution of 1.0 g/l nutrient combined with 100 mmol/L NaCl. After 25 days, we collected the aboveground biomass of the control and salt 100 mmol/L treatment, and the biomass of the nutrient limitation treatment 1 day later (Figure S1). The fresh weight was measured immediately. Subsequently, samples were dried for 3 days at 70°C after which dry weight was measured. We calculated the proportion of dry weight by dividing the dry weight by the fresh weight.

#### Greenhouse competition experiment

The greenhouse experiment including competition was performed in the summer of 2009. We used 90 RILs and the parent lines, aiming at 17 replicates per line per treatment. However, not all lines produced enough seedlings; so on average, 15.2 seedlings per line were analyzed, totaling up to 5594 plants that were used for this experiment over all treatments. Seeds were placed directly in the greenhouse in 4 by 4 cm pots with moist soil to induce germination. At the end of the establishment period, we reduced variation due to differential growth by removing the smallest and largest individuals, similarly as performed in the DR and SN experiment.

The competition experiment included of four treatments: control, nutrient limitation, salt, and drought. The germination and establishment period each lasted 9 days during which plants were shuffled once a week to prevent position effects. At the start of the stress period, pots were placed directly adjacent to each other (625 plants/m^2^) mimicking a strong intraspecific competition. Treatments were separated within the same greenhouse for logistic reasons. Within treatments, pots were placed randomly. The combined stress treatments were administered twice a week, identical to the SN and DR experiments. After 23 days of stress, we collected aboveground biomass of all treatments and measured dry weight as described above.

#### Field stress experiment

##### Field design

The field stress experiment was conducted in the same period as the greenhouse competition experiment during the summer of 2009. We used the same 90 RILs as in the competition experiment, with on average 13.5 seedlings per line (Table S1): totaling 3740 plants over all treatments. We recorded temperature and humidity levels in the field. The field site Sijbekarspel, The Netherlands (N52°42′, E04°58′) has a clay soil mimicking agricultural conditions with nutrient rich and high water retention conditions. We transplanted seedlings from the greenhouse to the field 9 days after sowing at the end of July 2009. In the field, we used a three-block design with a control, drought, and salt block. Within these blocks, plants were placed in a grid of 40 by 40 cm. Each block was subdivided into 17 subblocks, with 10 by 10 planting positions. This design allowed for each subblock to contain all RILs as well as the parental lines.

##### Stress experiments

At the start of the stress period, stress levels were increased gradually by applying the treatments daily for 4 days, after which treatments were administered three times a week. The control treatment was watered throughout the experiment. Drought stress was established by withholding water. The salt treatment was watered with a 100 mmol/L NaCl solution. After 21 days of stress, we collected aboveground biomass at the end of August 2009. Dry weight was assessed as described above.

### Statistical analysis

Statistical analyses were performed in PASW Statistics 17.0 (SPSS Inc. 2009). Testing for differences between parent lines within treatments was carried out with *t*-tests. All traits were transformed to improve normality of data distribution, prior to estimation of correlation across treatments, heritability values, and QTL analyses; biomass data were log-transformed and proportion data arcsine-square-root-transformed. Data distribution histograms are provided as Figure S3. Broad-sense heritability was estimated as the proportion of the total phenotypic variance accounted for by the genetic variation (Lynch and Walsh 1998). We estimated the correlation across treatments for biomass with the following equation (Lynch and Walsh 1998):


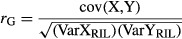


where Cov(X,Y) is the covariance of the average values of RILs in treatments X and Y, and VarX_RIL_ and VarY_RIL_ is among RIL variation for treatments X and Y, respectively, extracted with procedure VARCOMP (SPSS Inc. 2009). Note that the covariances and variances are estimated independently so that the estimates for the correlations can exceed plus or minus one. A high correlation indicates that the rank order of RILs is similar, that is, no Genotype x Environment interaction. Subsequently, the matrix of correlation estimates among treatments was used to build a clustering tree. To build this tree, we used the *hclust* function with Ward's method in R (version 2.14.0, R Development Core Team 2011). Prior to clustering, the correlation matrix was converted into a distances matrix using:


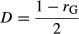


### QTL analysis

We performed QTL analysis on dry weight for all experiments and on proportion dry weight for the DR and SN experiments. Genetic map and marker data were obtained from The Compositae Genome Project website (http://compgenomics.ucdavis.edu). The genetic map we used consisted of 1513 markers distributed over the nine chromosomal linkage groups (http://cgpdb.ucdavis.edu/GeneticMapViewer/display/; map version: RIL_MAR_2007_ratio). All QTL analyses were performed with composite interval mapping (CIM) in QTL Cartographer version 2.5.008 (Wang et al. 2010). The analysis was performed at 2-cM intervals with a 10-cM window and five background cofactors that were selected both via forward and backward stepwise regression. Statistical significance threshold values (*α*=0.05) for declaring the QTL presence were estimated from 1000 permutations. One-LOD support intervals and additive effects were calculated from the CIM results. The linkage map and QTL positions were drawn with MapChart 2.2 (Voorrips 2002).

QTL were qualified as having a major effect (PVE > 25%), intermediate effect (PVE between 10–25%), or minor effect (PVE < 10%).

## Results

### Dry weight

#### Parental lines

We found significant differences in aboveground dry weight between the cultivated *L. sativa* cv. Salinas and the wild *L. serriola* parents in most greenhouse treatments. Exceptions were in the control of the salt/nutrient limitation (SN) experiment and in the combination of increased competition and nutrient limitation as well as drought (Table 1). In all field treatments, there was no significant difference between the parental lines in dry weight. As expected, within experiments, aboveground dry weight values were highest in the control treatments for both parental lines, with the only exception being the control dry weight of the crop parent in the greenhouse competition experiment.

#### RILs

For the RILs, broad-sense heritability values ranged from 17.0 to 65.5% (Table 1), with the lowest heritability found for plants grown under field conditions.

#### Qtl

**Figure 1:**
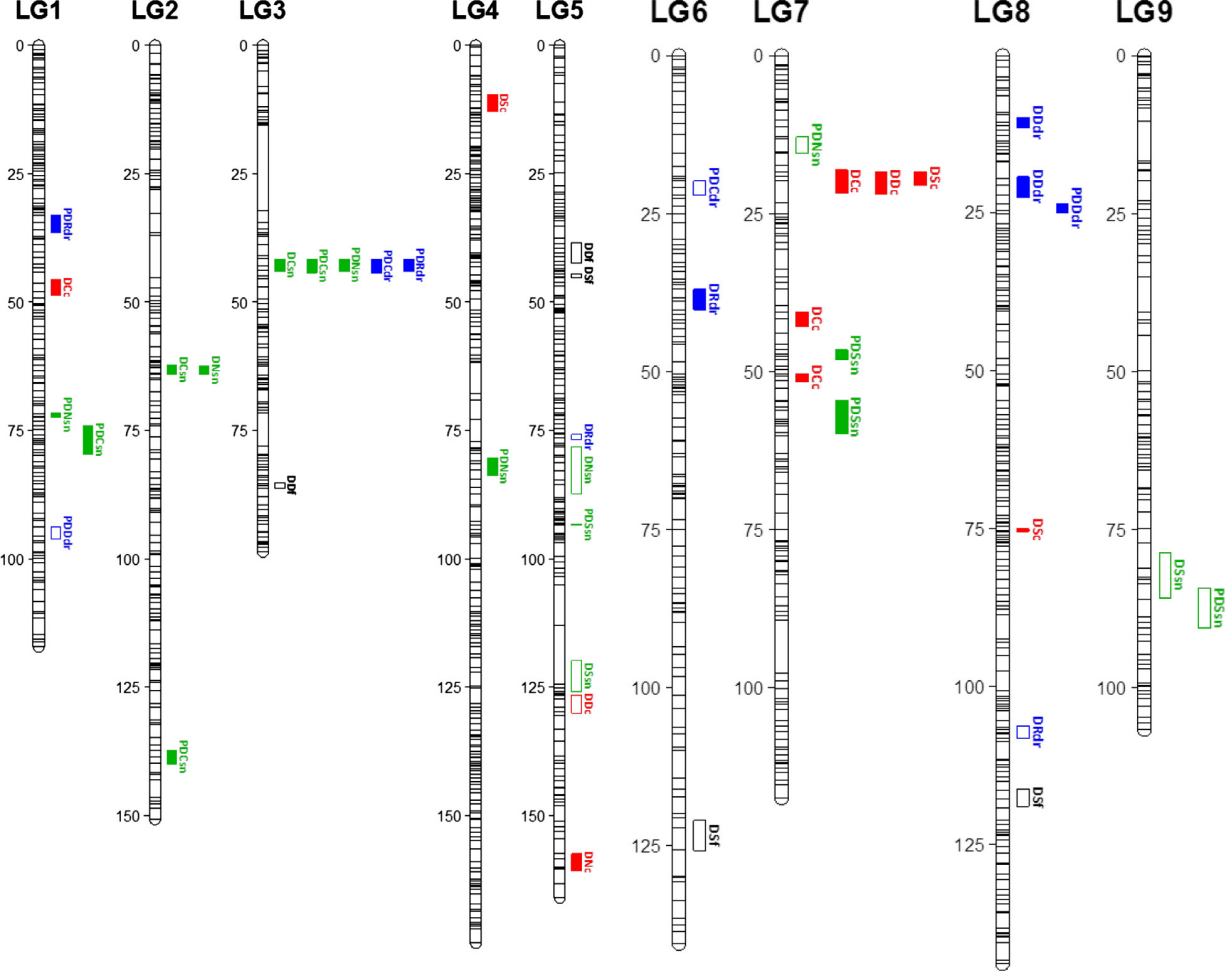
Genomic locations of QTL detected in composite interval mapping (CIM). Markers are indicated by horizontal lines on the linkage group bars and map distances (cM) are shown on the left side. Bars to the right represent one-LOD confidence intervals of QTL. An open bar indicates that the crop-type (*L. sativa* cv. Salinas) allele increases the trait values, whereas a filled bar indicates that the wild-type (*L. serriola*) allele increases the trait values. Bar colors indicate the experiment: Green, salt/nutrient limitation, Blue, drought/recovery, Red, Competition, and Black, Field. For abbreviations see Table 1.

We detected a total of 26 QTL for dry weight in 13 of 19 treatments, which were distributed over all nine linkage groups (Table 2; Fig. 1 with QTL abbreviations starting with “D”). The range of phenotypic variation explained (PVE) per QTL varied between 8.1 and 26.0%. The one-LOD support intervals were on average 3 cM (range 0.4–9.1 cM). In total, three (of 26) QTL were of major effect (PVE > 25% as defined by Burke et al. 2002): two major QTL were found for salt dry weight, one at LG5 in the field experiment and another at LG9 in the SN experiment. The third major QTL was found at LG3 for control dry weight in the SN experiment. Most of the 21 remaining QTL were of intermediate effect with only two QTL of minor effect.

**Table 2:** Composite interval mapping detected QTL in the *Lactuca sativa* cv. Salinas x *L. serriola* RIL population. Positive additive effects indicate that the crop-type (*L. sativa*) allele increases trait values, and negative additive effects indicate that the wild-type (*L. serriola*) allele increases trait values. Two major QTL clusters are indicated (LG3 or LG7)

Trait	LG	Position (cM)	1-LOD interval	Additive effect	PVE (%)	LOD	Threshold 0.05	Major cluster
Aboveground dry weight (g)								
Salt & nutrient limitation								
Control	2	62.7	62.3–64.1	−0.035	18	4.9	3.5	
Control	3	42.9	41.6–44.0	−0.042	25.4	6.6	3.5	LG3
Nutrient limitation	2	62.8	62.4–64.1	−0.028	13.8	4.1	3.5	
Nutrient limitation	5	79.9	78.2–87.3	0.03	17.5	5.2	3.5	
Salt 100 mmol/L	5	125.1	119.8–125.9	0.026	11.6	3.5	3.4	
Salt 100 mmol/L	9	81.2	78.7–85.8	0.041	26	6.7	3.4	
Increased drought (23 days) and recovery (4 days)								
Control	–							
Drought	8	10.6	10.0–11.6	−0.010	14.9	4.3	3.4	
Drought	8	20.7	19.4–22.6	−0.010	14.8	4.3	3.4	
Recovery	5	76.4	75.7–76.8	0.011	20.7	5.5	3.5	
Recovery	6	38.3	37.0–40.2	−0.010	15.8	4.7	3.5	
Recovery	8	106.6	106.3–108.2	0.011	19.9	4.7	3.5	
Increased competition								
Control	1	46.3	45.6–48.7	−0.025	8.1	3.6	3.5	
Control	7	19.2	18.2–21.6	−0.037	16.6	6.7	3.5	LG7
Control	7	41.7	40.6–42.9	−0.031	12.4	5.1	3.5	
Control	7	50.4	50.3–51.6	−0.029	11.2	4.8	3.5	
+ Drought	5	127.3	126.5–129.2	0.012	11.4	3.5	3.3	
+ Drought	7	19.2	18.5–21.7	−0.016	21.2	6.7	3.3	LG7
+ Nutrient limitation	5	158.4	157.4–161.0	−0.020	15.2	4.7	3.5	
+ Salt 100 mmol/L	4	10.9	9.6–12.9	−0.019	8.3	3.6	3.4	
+ Salt 100 mmol/L	7	19.2	18.4–20.6	−0.031	21.4	8.4	3.4	LG7
+ Salt 100 mmol/L	8	75.3	75.1–75.5	−0.027	17	7	3.4	
Field + stress								
Field only	–							
+ Drought	3	85.7	85.1–86.3	0.033	13	5	3.3	
+ Drought	5	42	38.4–42.4	0.039	18.5	6.5	3.3	
+ Salt 100 mmol/L	5	45.1	44.5–45.3	0.05	25.3	8.5	3.4	
+ Salt 100 mmol/L	6	122.2	121.0–125.8	0.033	10.6	4	3.4	
+ Salt 100 mmol/L	8	117.7	116.2–119.0	0.034	11.2	4	3.4	
Salt & nutrient limitation								
Control	1	74.8	74.0–79.7	−0.005	19.9	6.1	3.4	
Control	2	138.5	137.3–139.9	−0.005	20.6	6.7	3.4	
Control	3	42.9	41.6–44.3	−0.004	12.8	4.7	3.4	LG3
Nutrient limitation	1	72.4	71.5–72.4	−0.010	15.8	4.9	3.2	
Nutrient limitation	3	42.9	41.6–44.0	−0.012	19	6	3.2	LG3
Nutrient limitation	4	82.7	80.3–83.8	−0.009	9.9	3.5	3.2	
Nutrient limitation	7	13.2	12.9–15.5	0.01	14.1	4.6	3.2	LG7
Salt 100 mmol/L	5	93.5	93.2–93.5	0.005	13.9	4.7	3.4	
Salt 100 mmol/L	7	47.3	46.6–48.2	−0.005	15.7	4.4	3.4	
Salt 100 mmol/L	7	57.6	54.6–59.9	−0.006	22.5	6.9	3.4	
Salt 100 mmol/L	9	86.1	84.3–90.6	0.005	13.6	4.4	3.4	
Increased drought (23 days) and recovery (4 days)								
Control	3	42.9	41.6–44.4	−0.020	44.9	10.3	3.4	LG3
Control	6	20.8	19.9–22.1	0.011	17	4.9	3.4	
Drought	1	95.6	93.7–96.1	0.01	12.2	4	3.4	
Drought	8	24.5	23.6–25.1	−0.010	13.7	4.3	3.4	
Recovery	1	34.6	33.1–36.5	−0.006	14.3	4.1	3.4	
Recovery	3	42.9	41.6–44.0	−0.009	26.5	7	3.4	LG3

PVE, Percentage of variation explained.

### Proportion dry weight

#### Parental lines

In all SN and drought (DR) treatments, the wild parent had a higher aboveground proportion dry weight than the cultivated parent. This indicates the wild parent allocating more resources toward building up biomass and support tissue, whereas the crop parent produced broad leaves holding more water. Compared with the control treatments, the proportion dry weight of the parents increased under stress conditions. However, in the recovery treatment, the proportion dry weight returned to similar values as the control, indicating this allocation shift is reversible.

#### RILs

For the RILs, the proportion of dry weight showed higher heritability values than absolute dry weight measured in the same treatment, suggesting that the former is a trait related to the growth form of plants and less influenced by the environment. The only exception was the proportion dry weight of the control in the SN experiment (28.5%), which was caused by lower variability between lines compared with other treatments, leading to a low RIL variance compared with error variance.

#### Qtl

We detected a total of 17 QTL for aboveground dry weight in six treatments, distributed over all nine linkage groups (Table 2; Fig. 1 with QTL abbreviations starting with “PD”). PVE values per QTL varied between 9.9 and 44.9%. The one-LOD support intervals were on average 2.9 cM (range 0.3–9.1 cM). In total, two (of 17) QTL were of major effect: these colocalized at LG3 for both the control and recovery of the DR experiment. The majority of QTL (14) was of intermediate effect, with only one QTL of minor effect.

### QTL clusters

Overall, we detected 43 QTL for aboveground dry weight and proportion dry weight. Thirty-three QTL (76.7%) had a location that did not overlap with any other QTL (Fig. 1). The 10 QTL whose location did overlap with other QTL were located in the center of LG2 and LG3 and at the top of LG7. On LG2, only two QTL colocalized for the control and nutrient limitation of the SN experiment. On LG3, QTL were located for both dry weight and proportion dry weight of control and nutrient limitation treatments of the SN experiment, as well as control and recovery treatments of the DR experiment. On LG7, all three QTL were detected in the greenhouse competition experiment including dry weight QTL for control, drought and salt treatments. Notably, in all clusters, the wild allele (*L. serriola*) was correlated with an increased aboveground biomass and proportion of dry weight values. Although we detected a total of 38 QTL in the various greenhouse experiments, not one coincided with any of the five field QTL.

### Correlations between treatments

**Figure 2:**
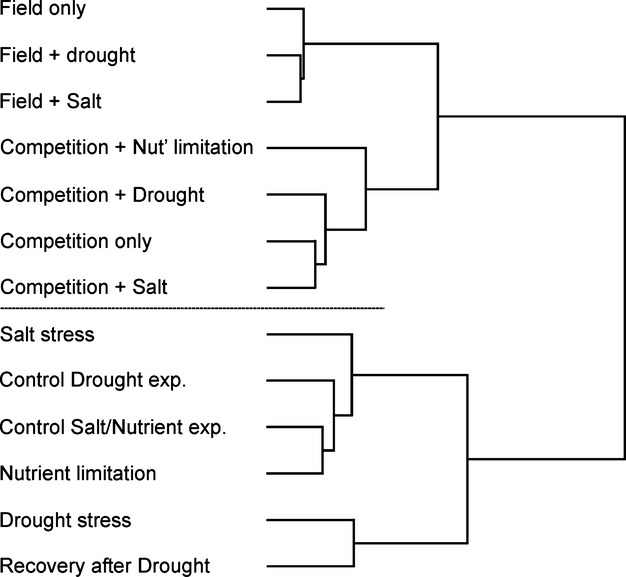
Tree-based clustering of treatments based on genetic correlations for biomass using their among treatment correlation distance matrix.

The highest correlations for biomass were generally between treatments of the same experiment (i.e., the same environment; Table 3): a strong correlation within experiments indicates a low Genotype x Environment (GxE) component. As depicted in the correlation tree of Fig. 2, treatments from the same experiment cluster together, the only exception being the control of the DR experiment, which clusters with the SN experiment. Furthermore, there is a clear split among experiments: the DR and SN experiments are placed in one branch of the tree, while the greenhouse competition and the field experiment are placed in a second branch (Fig. 2). The field treatments themselves showed relatively high correlations with each other (Table 3), suggesting that the added stress treatments in the field were not the major factors influencing the performance of the RILs. Rather, field conditions as such, as compared to greenhouse conditions, appeared to coincide with similar stress response.

**Table 3:** Correlation between treatments within and among environments using the VARCOMP procedure based on combined variance and covariance matrices. Note that the co-variances and variances were estimated independently so that the estimates for the correlations can exceed plus or minus one

	Greenhouse salt/nutrient			Greenhouse drought/recovery			Greenhouse competition				Field		
				
Experiment	Control	Salt 100 mmol/L	Nutrient limitation	Control	Drought	Recovery	Comp' only	Nutrient limitation	Drought	Salt 100 mmol/L	Field only	Drought	Salt 100 mmol/L
Greenhouse salt/nutrient													
Control	1.15	0.77	0.87	0.80	0.43	0.38	0.37	0.30	0.48	0.42	0.47	0.34	0.31
Salt 100 mmol/L		1.13	0.75	0.60	0.34	0.18	0.38	0.22	0.48	0.40	0.31	0.24	0.25
Nutrient limitation			1.09	0.82	0.42	0.08	0.61	0.45	0.61	0.67	0.64	0.54	0.51
Greenhouse drought/recovery													
Control				1.28	0.62	0.36	0.53	0.45	0.50	0.55	0.56	0.42	0.34
Drought					1.26	0.63	0.17	0.24	0.22	0.16	0.47	0.27	0.40
Recovery						1.68	−0.40	−0.05	−0.26	−0.37	0.09	−0.26	−0.16
Greenhouse competition													
Competition only							1.08	0.69	0.83	0.93	0.66	0.64	0.57
Nutrient limitation								1.05	0.63	0.64	0.58	0.46	0.60
Drought									1.15	0.91	0.69	0.68	0.64
Salt 100 mmol/L										1.06	0.68	0.73	0.62
Field													
Field only											1.28	1.04	1.02
Drought												1.41	1.05
Salt 100 mmol/L													1.37

## Discussion

The main result of our study is that various abiotic stress QTL were detected throughout the lettuce genome and generally did not colocalize, with the exception of only two clusters. We investigated whether there are general and consistent genomic locations of stress response on plant biomass across and within different experiments – that is, a few regions of major effect – or whether variability is high and QTL tend to comprise a mosaic across the genome. The results fit best to the latter hypothesis.

The overarching aim of our research is to identify regions within the genome that would, through selection in stressful environments, enhance or reduce the likelihood of introgression into populations of wild relatives. Unwanted crop genes located in these genomic regions would hitchhike along, and this would specifically be considered a problem for transgene events (Stewart et al. 2003). To provide a broader picture, in this discussion, we compare fitness impacts of the stress experiments to QTL found in earlier work within the same material but under uncontrolled “general field” conditions in which no specific stresses were added (Hartman et al. 2012, 2013a).

The only two clusters we detected in which three or more QTL co-occurred across experiments included mostly control treatment QTL and not the stress treatments themselves. Therefore, they are presumably more indicative for general growth. The two clusters are in the center of linkage group (LG) 3 and at the top of LG7. These regions coincide with QTL clusters identified in earlier experiments for morphological and general fitness-related traits (Hartman et al. 2012, 2013b; Uwimana et al. 2012a,b2012b). Therefore, we are confident that the major patterns we found in our experiments are not merely spurious correlations. For the first major cluster on LG3, five QTL from the greenhouse salt/nutrient limitation (SN) and drought/recovery (DR) experiments colocalized; four of which were QTL for proportion dry weight of SN and DR controls and nutrient limitation and drought recovery treatments. The presence of these control treatment QTL suggests that this genomic region presents a general growth response among RILs rather than a stress response. Furthermore, this region is known to be correlated with QTL for leaf shape and seed output traits (Hartman et al. 2013b). We suggest that the combination of the growth response and this earlier found leaf shape QTL indicates that the transition from narrow wild-type leaves to broad cultivated type leaves has coincided with a reduction in support tissues and an increase in water contents of the leaves of the crop (de Vries 1997). This transition would have affected the proportion of aboveground dry weight in the leaves. For the second major cluster, which is located on LG7, it was striking that all QTL were from the greenhouse competition experiment and included the control, drought, and salt stress QTL. This region is known from earlier work to be correlated with the speed of development and is most likely governed by a common major gene for earliness of flowering (Hartman et al. 2013b). The implication of the combination of our results and this suggested “speed-of-development gene” is further discussed below.

Next to those two clusters, we found numerous non-overlapping stress QTL, some of which were even different among experiments that included the same stress. One reason could be that different abiotic stresses could cause variable genetic expression patterns. This is, for example, supported by the low correlation between the salt and drought treatments of the greenhouse stress experiments. A plant's response to abiotic stresses involves complex signaling pathways, depending on many genes, proteins, and metabolic pathways that may also vary across life stages (Knight and Knight 2001; Roy et al. 2011). This pathway complexity could introduce an element of chance in pinpointing stress response location, as cascading genes could be spread across the genome. However, such draw-backs need to be weighed against the advantages of correlative studies such as QTL that provide an overall assessment of the whole genome as a merely expectation-free bottom-up approach. Alternative techniques such as knock-out mutants would focus on specific genes as a top (researcher)-down approach and hence are more mechanistically and preknowledge driven.

The second reason that could generate variation among experiments is unavoidable variability in the applied stresses across experiments. Although we aimed to apply stresses as similar as possible in the various experiments, some differences in design and conditions were unavoidable. Those differences included substrate differences for the salt and nutrient addition experiments combined with full substrate flushing, the length of the stress periods, and the progressing time of year for serial experiments. As a consequence, the exact amount and timing of applied stress observed by the plants could have differed among the experiments. Indeed, genetic correlations between different abiotic stress treatments within the same experiments were high, whereas those between treatments for a specific stress across different experiments were low, indicating a high Genotype by Environment (GxE) interaction between experiments. This implies that it is likely difficult to design and perform a set of experiments that consistently determine the QTL for a particular abiotic stress, because necessary changes in the setup, such as differences in plant age and initial growing conditions, may already cause different expression patterns in response to stress (Collins et al. 2008).

Alternatively, it could be criticized that there was not enough statistical power due to a small sample size and low number of replicates (necessitated by the scale of the experiment), even though we used 11,380 plants in this study. Low power could lead to low heritability values, leaving QTL undetected (Beavis 1998; Mauricio 2001; Collard et al. 2005). Indeed, field heritability values were lower than in the greenhouse due to a higher environmental variation (Gardner and Latta 2008), which was not fully countered by a higher number of replicates used in the field. However, heritability values were >50% in the majority of greenhouse treatments, with the exception of the drought treatment. Such high heritabilities indicate a substantial genetic component underlying the variation and so a good ability to locate QTL (Hyne et al. 1995). As the two major clusters identified are the same as those found in separate studies (Hartman et al. 2012, 2013a,b2013b), we consider it unlikely that other major QTL locations correlated with the measured traits would have gone unnoticed.

### Outrunning competitors

Our results suggest that genomic segments from the wild species, *L. serriola*, make hybrid plants better competitors compared with the respective cultivated genomic segments. In the greenhouse competition experiments, wild genomic segments at those QTL induced a higher aboveground biomass, as seen in the major cluster of multiple competition QTL at the top of LG7. In an earlier experiment, under not-specified general field conditions, Hartman et al. (2012, 2013a) followed plants through their entire life cycle. They detected fitness QTL at this same genomic location as well as several QTL connected to the speed of development (Hartman et al. 2013b). Now using a much more controlled experiment with single stresses, we have more insights into the mechanism: the wild allele seems to induce a higher speed-of-development at this genomic location: faster growth, early bolting, and hence flowering. This suggests that under such competitive circumstances with high plant density, it is selectively advantageous to have a faster development, produce more biomass quickly and to bolt earlier in order to outrun the competitors (Fakheran et al. 2010). Our evidence is further supported by a second set of observations made in full life-cycle experiments under “general” field condition in which *L. sativa* and plants with a morphology closer to *L. sativa* died before reproduction (Hooftman et al. 2005, 2007).

In general, the timing of bolting and flowering influences the ability of crop–wild hybrids to survive and produce biomass. Radish, which is a crop bred for its vegetative parts just as lettuce, also has a delayed flowering time compared with its wild relative. In crop–wild radish hybrids, a decline in white flower color, a dominant crop allele linked to delayed flowering time, was observed after a decade of following crop allele frequencies in experimental competitively selective populations (Campbell and Snow 2007; Campbell et al. 2009; Snow et al. 2010). For both lettuce and radish, it is known that hybridization can produce vigorous crop–wild hybrids that were interpreted to result from new additive genetic combinations leading to increased fitness and (potential) competitiveness (Hooftman et al. 2009, 2011; Snow et al. 2010). These studies on lettuce and radish suggest that, under high population densities – as in our competition experiment – the wild genomic background conferring early flowering at specific genomic locations increases the competing ability of crop–wild hybrids.

It could be argued that oppositely late flowering might be more advantageous in those cases where the environment would allow an extended flowering period. The increased biomass due to a longer growth period would result in larger plants with presumably more shoots and branches and, eventually, more seed output. In addition, plant densities can be highly variable in wild populations. Follow-up experiments in situations with low plant density but an extended flowering period could be initiated to determine whether in that situation the wild genomic background would increase the competitive ability of crop–wild hybrids. Irrespective of such low density situations, in situations with high plant density, as we tested here, and with a seasonal flowering period, lettuce hybrids with a crop genomic background at LG7 will have a higher likelihood to be outcompeted by their wild relatives and die before reproduction.

## Implications for GM Environmental Risk Assessment

Controlled, short-term greenhouse experiments are often used to evaluate the effect of transgenes, rather than observing the effects of stress during the life span of a crop in a range of agricultural conditions (Vinocur and Altman 2005; Bhatnagar-Mathur et al. 2008; Mittler and Blumwald 2010). Our results indicate that a series of short-term experiments are not necessarily sufficient or fully informative to determine the likelihood of permanent introgression of unwanted crop genes in wild relative populations based on background genomic selection patterns. Unwanted crop genes may include potential transgenes (GMOs). Variation in QTL expression patterns was relatively low in the case where multiple stresses are applied to plants in a single all-including experimental setup at the same time and place; such was performed in, for example, Uwimana et al. (2012a,b2012b). However, mostly this is not feasible, and multiple or serial experimental setups need to be used that could include combinations of indoor and outdoor experiments. In our study, we carried out such experiments, and we found little overlap in QTL between such controlled greenhouse experiments and the field situation, except for competition. The genetic correlations in our study indicated that the greenhouse competition treatments had the highest correlation with the field treatments, suggesting that competition rather than a specific abiotic stress was an important influential factor in the field. In addition, predictions from a particular study only hold as long as selection pressures are similar. The strong GxE interactions we found here might imply that the genomic regions that could come under selection might be broader or more variable than was thought earlier (Hooftman et al. 2011; Hartman et al. 2012, 2013a) and could depend on specific conditions in the field (Weinig et al. 2002; Martin et al. 2006). Therefore, it will be very difficult to predict which genetic regions from the crop might contain a selective advantage after escape into neighboring wild relative populations in different environments.

On a more positive note, this study strengthens earlier work by Hartman, Uwimana, and coworkers (Hartman et al. 2012, 2013a,b2013b; Uwimana et al. 2012b,c2012c) in consistently pinpointing two major genomic regions with similar species-directional selection effects across various field and greenhouse environments. Despite the variability on other regions, these two specific regions could be used in transgene mitigation (TM) strategies (Gressel 1999; Stewart et al. 2003). The rationale for such a strategy is that a transgene located in a genomic region that is selected against in the wild is more likely to be purged from the wild population (Stewart et al. 2003). TM strategies were successfully tested by placing a transgene in linkage with a dwarfing gene in tobacco and oil seed rape (Al-Ahmad et al. 2005; Rose et al. 2009). The results from our study could indicate that selection for a delay in bolting and flowering, genomic regions coding for delay in or even prevention of flowering might result in a few good candidates for such TM strategies. The two major blocks with a strong preference for the wild allele would fit the requirements of such region.

In conclusion, unavoidable differences in experimental setup can cause a large variation in QTL results, making predicting genomic selection patterns for specific abiotic stress environments challenging. Therefore, considerable efforts would be required in terms of plants, lines, and manual labor in order to include genomic location to specific abiotic stresses as proxy for the likelihood of introgression into wild relative populations, in ERA (Beavis 1998). On the positive side, selection in response to increased competition seems to provide a much more general genomic location signal. Therefore, we would recommend for risk assessment experiments to preferably include multiple plant densities and multispecies environments for estimations of the likelihood of introgression of transgenes in wild relative populations. In this recommendation we align with studies in other crops (e.g., Mercer et al. 2006; Campbell and Snow 2007; Ellstrand et al. 2013).

## Supporting Information

**Table S1.** Number of replicates per line used in the greenhouse competition and field experiments. **Figure S1.** Schematic representation of experimental design. **Figure S2.** (a) Water capacity of empty pots during the stress period of the drought/recovery experiment. (b) Electric conductivity of empty pots during the stress period. Control and nutrient limitation treatments are on the left axis and the salt treatments are on the right axis. **Figure S3.** Data distributions of input data for the QTL analyses. **Appendix S1.** Environmental conditions during the greenhouse experiments.
