# Supplementary material for: Abiotic stress QTL in lettuce crop–wild hybrids: comparing greenhouse and field experiments
Source: Ecol Evol. 2014 May 17;4(12):2395–409. doi: 10.1002/ece3.1060 (PMC4203288; doi:10.1002/ece3.1060)
Supplement: Supplementary file 1 [file ece30004-2395-sd1.docx]

**Supplementary information for**

**Hartman et al.**

**Abiotic stress QTL in lettuce crop–wild hybrids: comparing greenhouse and field experiments**

**Table S1.** Number of replicates per line used in the greenhouse competition and field experiments**.**

**Figure S1.** Schematic representation of experimental design.

**Figure S2**. Water capacity of empty pots during the stress period of the Drought/Recovery experiment.

**Figure S2b**. Electric conductivity of empty pots during the stress period.

**Figure S3.** Data distributions of input data for the QTL analyses.

**Appendix S1.** Environmental conditions during the greenhouse experiments.

**Table S1.** Number of replicates per line used in the greenhouse competition and field experiments**.**

|  | **Greenhouse** | | | | **Field** | | |
| --- | --- | --- | --- | --- | --- | --- | --- |
| **RIL** | **Control** | **Nutrient limitation** | **Drought** | **Salt 100mM** | **Control** | **Drought** | **Salt 100mM** |
| *L. sativa* cv. Salinas | 17 | 17 | 17 | 17 | 17 | 14 | 10 |
| *L. serriola* UC96US23 | 17 | 17 | 17 | 17 | 13 | 10 | 12 |
| 114 | 17 | 17 | 17 | 17 | 17 | 14 | 14 |
| 115 | 17 | 0 | 17 | 17 | 17 | 14 | 13 |
| 116 | 17 | 17 | 17 | 17 | 15 | 14 | 13 |
| 119 | 12 | 15 | 13 | 11 | 11 | 10 | 10 |
| 120 | 17 | 17 | 17 | 17 | 16 | 14 | 14 |
| 121 | 7 | 17 | 7 | 7 | 17 | 13 | 16 |
| 122 | 17 | 17 | 17 | 17 | 16 | 16 | 16 |
| 124 | 14 | 17 | 16 | 15 | 14 | 11 | 7 |
| 125 | 14 | 17 | 16 | 17 | 17 | 15 | 15 |
| 126 | 2 | 17 | 3 | 3 | 14 | 12 | 16 |
| 127 | 17 | 7 | 17 | 17 | 17 | 16 | 13 |
| 128 | 13 | 8 | 13 | 13 | 9 | 7 | 11 |
| 129 | 17 | 17 | 17 | 17 | 16 | 15 | 14 |
| 130 | 17 | 17 | 17 | 17 | 16 | 13 | 12 |
| 131 | 14 | 17 | 14 | 17 | 7 | 7 | 7 |
| 132 | 9 | 17 | 10 | 10 | 17 | 15 | 16 |
| 133 | 10 | 16 | 10 | 10 | 12 | 9 | 8 |
| 134 | 17 | 17 | 17 | 17 | 16 | 14 | 16 |
| 135 | 17 | 16 | 17 | 17 | 16 | 14 | 15 |
| 136 | 15 | 17 | 15 | 16 | 9 | 10 | 14 |
| 139 | 17 | 17 | 17 | 17 | 17 | 13 | 11 |
| 140 | 9 | 17 | 10 | 11 | 14 | 13 | 8 |
| 141 | 9 | 17 | 9 | 9 | 9 | 9 | 6 |
| 142 | 12 | 17 | 12 | 13 | 17 | 14 | 17 |
| 143 | 17 | 17 | 15 | 16 | 17 | 15 | 12 |
| 144 | 17 | 17 | 17 | 17 | 15 | 14 | 16 |
| 145 | 3 | 13 | 3 | 4 | 10 | 7 | 8 |
| 146 | 17 | 17 | 17 | 17 | 15 | 16 | 15 |
| 148 | 17 | 17 | 17 | 17 | 9 | 8 | 9 |
| 149 | 17 | 17 | 17 | 17 | 17 | 14 | 15 |
| 150 | 13 | 17 | 14 | 13 | 17 | 11 | 17 |
| 151 | 17 | 17 | 17 | 17 | 16 | 14 | 14 |
| 152 | 17 | 17 | 17 | 17 | 16 | 15 | 16 |
| 154 | 17 | 17 | 17 | 17 | 15 | 12 | 14 |
| 155 | 16 | 17 | 14 | 16 | 15 | 15 | 13 |
| 156 | 17 | 17 | 17 | 17 | 15 | 14 | 13 |
| 158 | 11 | 5 | 12 | 12 | 9 | 7 | 5 |
| 159 | 14 | 17 | 15 | 15 | 17 | 12 | 14 |
| 160 | 17 | 17 | 17 | 17 | 15 | 16 | 16 |
| 162 | 17 | 17 | 17 | 17 | 16 | 14 | 15 |
| 163 | 10 | 17 | 9 | 9 | 14 | 14 | 11 |
| 164 | 17 | 17 | 17 | 17 | 17 | 15 | 16 |
| 166 | 17 | 17 | 17 | 17 | 16 | 16 | 14 |
| 167 | 13 | 17 | 13 | 13 | 17 | 14 | 13 |
| 169 | 13 | 14 | 13 | 13 | 10 | 0 | 0 |
| 170 | 17 | 17 | 17 | 17 | 17 | 15 | 13 |

**Table S1.** Continued

|  | **Greenhouse** | | | | **Field** | | |
| --- | --- | --- | --- | --- | --- | --- | --- |
| **RIL** | **Control** | **Nutrient limitation** | **Drought** | **Salt 100mM** | **Control** | **Drought** | **Salt 100mM** |
| 171 | 17 | 17 | 17 | 17 | 17 | 13 | 14 |
| 172 | 15 | 17 | 15 | 16 | 17 | 15 | 15 |
| 173 | 9 | 17 | 8 | 8 | 6 | 5 | 4 |
| 175 | 17 | 11 | 17 | 17 | 12 | 14 | 13 |
| 176 | 17 | 17 | 17 | 17 | 16 | 13 | 12 |
| 177 | 17 | 17 | 17 | 17 | 16 | 14 | 11 |
| 178 | 17 | 17 | 17 | 17 | 14 | 13 | 10 |
| 179 | 12 | 15 | 12 | 11 | 13 | 14 | 14 |
| 180 | 17 | 17 | 17 | 17 | 17 | 16 | 11 |
| 181 | 8 | 4 | 8 | 11 | 14 | 8 | 10 |
| 182 | 17 | 17 | 17 | 17 | 17 | 16 | 16 |
| 183 | 17 | 17 | 17 | 17 | 12 | 11 | 12 |
| 186 | 17 | 17 | 17 | 17 | 15 | 15 | 15 |
| 187 | 13 | 15 | 13 | 14 | 15 | 14 | 13 |
| 190 | 17 | 17 | 17 | 17 | 16 | 15 | 13 |
| 192 | 15 | 13 | 17 | 17 | 17 | 17 | 14 |
| 193 | 17 | 17 | 17 | 17 | 16 | 15 | 12 |
| 194 | 17 | 17 | 17 | 17 | 16 | 13 | 15 |
| 195 | 17 | 17 | 17 | 17 | 16 | 14 | 12 |
| 196 | 17 | 17 | 17 | 17 | 13 | 14 | 15 |
| 197 | 17 | 16 | 17 | 17 | 15 | 14 | 12 |
| 199 | 10 | 14 | 16 | 16 | 16 | 11 | 13 |
| 201 | 17 | 17 | 17 | 17 | 16 | 16 | 14 |
| 202 | 17 | 10 | 17 | 17 | 16 | 16 | 16 |
| 203 | 17 | 17 | 17 | 17 | 13 | 11 | 14 |
| 204 | 17 | 17 | 17 | 17 | 16 | 14 | 15 |
| 205 | 13 | 17 | 17 | 17 | 15 | 14 | 11 |
| 206 | 15 | 17 | 15 | 15 | 16 | 15 | 17 |
| 207 | 17 | 14 | 17 | 17 | 16 | 15 | 16 |
| 209 | 17 | 17 | 17 | 17 | 17 | 15 | 14 |
| 211 | 17 | 17 | 17 | 17 | 15 | 15 | 10 |
| 212 | 17 | 17 | 17 | 17 | 17 | 14 | 13 |
| 213 | 17 | 11 | 17 | 17 | 15 | 14 | 12 |
| 214 | 12 | 17 | 12 | 12 | 9 | 10 | 9 |
| 215 | 14 | 15 | 14 | 14 | 13 | 12 | 9 |
| 217 | 14 | 12 | 14 | 15 | 15 | 13 | 16 |
| 218 | 12 | 17 | 12 | 12 | 17 | 16 | 16 |
| 219 | 17 | 17 | 17 | 17 | 16 | 11 | 14 |
| 220 | 16 | 17 | 17 | 16 | 17 | 14 | 14 |
| 221 | 15 | 17 | 15 | 15 | 15 | 13 | 15 |
| 222 | 17 | 17 | 17 | 17 | 16 | 11 | 14 |
| 223 | 13 | 17 | 11 | 12 | 13 | 14 | 14 |
| 227 | 12 | 14 | 13 | 13 | 16 | 14 | 13 |
| 228 | 14 | 9 | 15 | 12 | 10 | 11 | 11 |
| **average n treatment** | 14.8 | 15.7 | 15.0 | 15.1 | 14.7 | 13.0 | 12.8 |
| **average n experiment** | 15.2 | | | | 13.5 | | |

**Figure S1.** Schematic representation of experimental design. Blue = germination period, green = establishment period, orange = stress period, purple = biomass collection, and dark blue = rewater period. 1 = drought treatment collected, 2 = control and recovery treatment collected.

| Week | **1** | | | | | | | **2** | | | | | | | **3** | | | | | | | **4** | | | | | | | **5** | | | | | | | **6** | | | | | | | **7** | | | | | | |
| --- | --- | --- | --- | --- | --- | --- | --- | --- | --- | --- | --- | --- | --- | --- | --- | --- | --- | --- | --- | --- | --- | --- | --- | --- | --- | --- | --- | --- | --- | --- | --- | --- | --- | --- | --- | --- | --- | --- | --- | --- | --- | --- | --- | --- | --- | --- | --- | --- | --- |
| Salt/ Nutrient limitation |  |  |  |  |  |  |  |  |  |  |  |  |  |  |  |  |  |  |  |  |  |  |  |  |  |  |  |  |  |  |  |  |  |  |  |  |  |  |  |  |  |  |  |  |  |  |  |  |  |
| Drought/  Recovery |  |  |  |  |  |  |  |  |  |  |  |  |  |  |  |  |  |  |  |  |  |  |  |  |  |  |  |  |  |  |  |  |  |  |  |  |  |  |  |  |  |  | **1** |  |  |  | **2** |  |  |
| Greenhouse  competition |  |  |  |  |  |  |  |  |  |  |  |  |  |  |  |  |  |  |  |  |  |  |  |  |  |  |  |  |  |  |  |  |  |  |  |  |  |  |  |  |  |  |  |  |  |  |  |  |  |
| Field stress |  |  |  |  |  |  |  |  |  |  |  |  |  |  |  |  |  |  |  |  |  |  |  |  |  |  |  |  |  |  |  |  |  |  |  |  |  |  |  |  |  |  |  |  |  |  |  |  |  |

**Figure S2a**. Water capacity of empty pots during the stress period of the Drought/Recovery experiment.

**Figure S2b**. Electric conductivity of empty pots during the stress period. Control and nutrient limitation treatments are on the left axis and the salt treatments are on the right axis.

**Figure S3.** Data distributions of input data for the QTL analyses. Shown are the transformed data as used as input for QTL cartographer in histograms of 10 bars for all treatments as presented in Table 1-3 and Figures 1 and 2. Intervals between bars are given and are different for every treatments.

Dry weight of aboveground biomass of the Greenhouse salt & nutrient experiment: a) control treatment; b) Added salt of 100mM; c) Nutrient limitation (no addition).

Dry weight of aboveground biomass of the Greenhouse Drought experiment: d) control treatment; e) 23 days of drought; f) 4 days of recovery after drought.

Dry weight of aboveground biomass of the Greenhouse Competition experiment: g) increased competition (high density) only; h) increased competition and nutrient limitation; i) increased competition and added salt 100mM; j) increased competition and 23 days of drought.

Dry weight of aboveground biomass of the Field experiment: k) general field conditions only; l) general field conditions and Salt added 100mM; m) general field conditions and Drought.

Proportion Dry weight of fresh biomass of the Greenhouse salt & nutrient experiment: n) control treatment; o) Added salt of 100mM; p) Nutrient limitation (no addition).

Proportion Dry weight of fresh biomass of the Greenhouse Drought experiment: q) control treatment; r) 23 days of drought; s) 4 days of recovery after drought.

**Fig S3.**

**Fig S3 (continued).**

**Fig S3 (continued).**

**Appendix S1.** Environmental conditions during the greenhouse experiments.

For both the Drought/recovery experiment and the Salt/nutrient limitation experiment, treatment conditions were stable throughout the stress period (See Supplementary material Figure S2). In the Drought experiment temperatures ranged from 17.9°C to 22.5°C, with an average temperature of 19.6°C and relative humidity of 77.8%. In the SN experiment temperatures ranged from 17.9°C to 25.4°C, with an average of 21.3°C and relative humidity of 61.9%. In the field experiment the average temperature was 19.8°C and relative humidity was 73.5% during the establishment and stress period in July and August 2009. The maximum temperature reached 36.6°C and a minimum of 7.8°C. During the stress period there was no precipitation, providing good conditions for the drought stress treatment.
